# Effect of a Lifestyle-Focused Web-Based Application on Risk Factor Management in Patients Who Have Had a Myocardial Infarction: Randomized Controlled Trial

**DOI:** 10.2196/25224

**Published:** 2022-03-31

**Authors:** Halldóra Ögmundsdóttir Michelsen, Ingela Sjölin, Maria Bäck, Manuel Gonzalez Garcia, Anneli Olsson, Camilla Sandberg, Alexandru Schiopu, Margrét Leósdóttir

**Affiliations:** 1 Department of Clinical Sciences Malmö Lund University Malmö Sweden; 2 Department of Internal Medicine Helsingborg Hospital Helsingborg Sweden; 3 Department of Cardiology Skane University Hospital Malmö Sweden; 4 Department of Health, Medicine and Caring Sciences, Unit of Physiotherapy Linköping University Linköping Sweden; 5 Department of Occupational Therapy and Physiotherapy Sahlgrenska University Hospital Gothenburg Sweden; 6 Department of Epidemiology and Global Health Umeå University Umeå Sweden; 7 Faculty of Medicine The University of Queensland Herston, Queensland Australia; 8 Department of Cardiology Skane University Hospital Lund Sweden; 9 Department of Clinical Sciences Lund University Lund Sweden; 10 Department of Community Medicine and Rehabilitation, Physiotherapy Umeå University Umeå Sweden; 11 Department of Internal Medicine Skane University Hospital Lund Sweden

**Keywords:** eHealth, cardiac rehabilitation, cardiovascular, mobile device app, risk factors, web-based application, mobile phone

## Abstract

**Background:**

Cardiac rehabilitation is central in reducing mortality and morbidity after myocardial infarction. However, the fulfillment of guideline-recommended cardiac rehabilitation targets is unsatisfactory. eHealth offers new possibilities to improve clinical care.

**Objective:**

This study aims to assess the effect of a web-based application designed to support adherence to lifestyle advice and self-control of risk factors (intervention) in addition to center-based cardiac rehabilitation, compared with cardiac rehabilitation only (usual care).

**Methods:**

All 150 patients participated in cardiac rehabilitation. Patients randomized to the intervention group (n=101) received access to the application for 25 weeks where information about lifestyle (eg, diet and physical activity), risk factors (eg, weight and blood pressure [BP]), and symptoms could be registered. The software provided feedback and lifestyle advice. The primary outcome was a change in submaximal exercise capacity (Watts [W]) between follow-up visits. Secondary outcomes included changes in modifiable risk factors between baseline and follow-up visits and uptake and adherence to the application. Regression analysis was used, adjusting for relevant baseline variables.

**Results:**

There was a nonsignificant trend toward a larger change in exercise capacity in the intervention group (n=66) compared with the usual care group (n=40; +14.4, SD 19.0 W, vs +10.3, SD 16.1 W; *P*=.22). Patients in the intervention group achieved significantly larger BP reduction compared with usual care patients at 2 weeks (systolic −27.7 vs −16.4 mm Hg; *P*=.006) and at 6 to 10 weeks (systolic −25.3 vs −16.4 mm Hg; *P*=.02, and diastolic −13.4 vs −9.1 mm Hg; *P*=.05). A healthy diet index score improved significantly more between baseline and the 2-week follow-up in the intervention group (+2.3 vs +1.4 points; *P*=.05), mostly owing to an increase in the consumption of fish and fruit. At 6 to 10 weeks, 64% (14/22) versus 46% (5/11) of smokers in the intervention versus usual care groups had quit smoking, and at 12 to 14 months, the respective percentages were 55% (12/22) versus 36% (4/11). However, the number of smokers in the study was low (33/149, 21.9%), and the differences were nonsignificant. Attendance in cardiac rehabilitation was high, with 96% (96/100) of patients in the intervention group and 98% (48/49) of patients receiving usual care only attending 12- to 14-month follow-up. Uptake (logging data in the application at least once) was 86.1% (87/101). Adherence (logging data at least twice weekly) was 91% (79/87) in week 1 and 56% (49/87) in week 25.

**Conclusions:**

Complementing cardiac rehabilitation with a web-based application improved BP and dietary habits during the first months after myocardial infarction. A nonsignificant tendency toward better exercise capacity and higher smoking cessation rates was observed. Although the study group was small, these positive trends support further development of eHealth in cardiac rehabilitation.

**Trial Registration:**

ClinicalTrials.gov NCT03260582; https://clinicaltrials.gov/ct2/show/NCT03260582

**International Registered Report Identifier (IRRID):**

RR2-10.1186/s13063-018-3118-1

## Introduction

### Background

Mortality rates from coronary heart disease (CHD) have decreased in the last decades [1-3]. However, the falling mortality rates have led to an increased number of survivors who need support in the secondary prevention of recurrent coronary events [3,4]. Comprehensive cardiac rehabilitation programs, which are multidisciplinary medical and health behavioral interventions, effectively reduce CHD morbidity and mortality [3-7]. International guidelines strongly recommend participation in cardiac rehabilitation and have set therapeutic goals on risk factor and lifestyle management [7]. Cardiac rehabilitation goals include smoking cessation, medical management of low-density lipoprotein (LDL)-cholesterol and blood pressure (BP), maintaining a healthy diet, and participation in an exercise training program, also referred to as exercise-based cardiac rehabilitation [7]. Despite clear set goals, both attendance to cardiac rehabilitation and therapeutic goal attainment are suboptimal [8,9].

Cardiac rehabilitation programs in Sweden hold a high standard compared with European counterparts when it comes to providing guideline-recommended services [10]. Moreover, in 2019, the Swedish Web-System for Enhancement and Development of Evidence-Based Care in Heart Disease Evaluated According to Recommended Therapies (SWEDEHEART) registry reported that after participating in a 1-year long cardiac rehabilitation program, patients who had an myocardial infarction (MI) only reached target levels for LDL-cholesterol in about 60% of cases, only 55% of smokers were abstinent and 19% of patients had participated in exercise-based cardiac rehabilitation programs [9]. A lack of reaching therapeutic goals while offering guideline-recommended services indicates that alternative methods may be needed to improve patient outcomes.

Previous studies on eHealth cardiac rehabilitation have resulted in noninferiority or concluded that patients benefited with regard to lifestyle changes and risk factor management [11-20]. However, these studies have varied in sample size and follow-up time, and more studies are needed. eHealth is of interest to the field of cardiac rehabilitation because it has the potential to overcome some known barriers to participation, such as geographical distance, communication barriers, and rigid follow-up structures, and to individualize cardiac rehabilitation programs [4,21].

### Objectives

The aim of this study is to evaluate the efficacy of a web-based application as an addition to a comprehensive center-based cardiac rehabilitation program, in comparison with usual care center-based cardiac rehabilitation only. The web-based application was designed to support adherence to lifestyle advice and self-control of risk factors in patients who had an MI. In the planning phase of our study, we predicted that the web-based application would primarily affect patients’ lifestyle and with that increased physical activity levels and exercise capacity [22]. The benefits of increased physical activity levels and participation in exercise-based cardiac rehabilitation are largely mediated through an increase in physical fitness. Submaximal exercise capacity is an objective measurement of physical fitness and was, therefore, chosen as the primary outcome [23].

## Methods

### Trial Design and Framework

The protocol, which followed the principles outlined in the Declaration of Helsinki, was approved by the Regional Ethical Review Board at Lund University (approval 2016/5). The protocol has been previously described in detail [22] and is summarized here.

We conducted an unblinded parallel multicenter randomized controlled trial (RCT) at 3 cardiac rehabilitation centers based at university hospitals in Sweden. At the time of the study, approximately 1200 patients aged <75 years were treated for acute MI at the 3 study centers each year.

### Participants, Recruitment, and Randomization

The inclusion criteria were age 18-74 years, having had an MI within the last 2 weeks, owning a smartphone or having access to the internet via a computer or tablet, and being able to handle the software. The exclusion criteria were having an expected survival of <1 year, dementia, severe psychiatric illness or drug abuse, severe physical disability limiting the patients’ ability to participate in a center-based exercise-based cardiac rehabilitation, inability to speak or understand the Swedish language, and a 3-vessel or left main disease requiring coronary artery bypass grafting. The age criterion was set to match that applied by the SWEDEHEART registry.

Eligibility screening and study inclusion were performed within 2 weeks of an index MI while participants were admitted to a coronary care unit at each participating hospital. Local study coordinators (physicians, nurses, or physiotherapists) provided eligible patients with information about the study, offered participation, and obtained written informed consent. Once included in the study, the participants were randomized to 1 of the 2 study arms using opaque sealed envelopes. The envelopes, which included information on which study group the participant would be randomized to, were prepared by a member of the research team and shuffled by another member. The envelopes were thereafter sequentially numbered with unique numbers for each recruiting site. Upon recruitment, baseline questionnaires were administered.

### Usual Care

**Figure 1:**
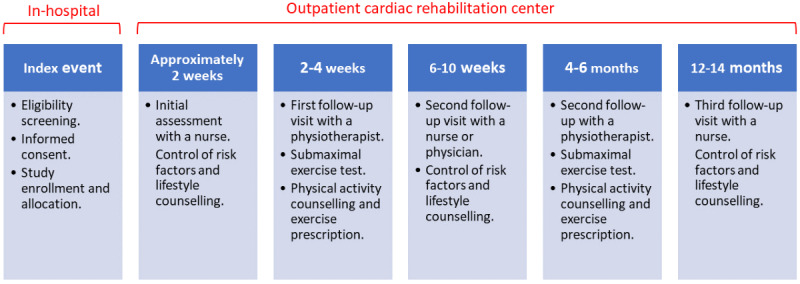
The follow-up of the patients who have had a myocardial infarction, in the study.

Participants in both arms of the study were offered participation in a comprehensive cardiac rehabilitation program at each center. A description of the SPIRIT (Standard Protocol Items: Recommendations for Interventional Trials) figure on the content of each follow-up visit is available in the protocol [22]. In short, the cardiac rehabilitation programs consisted of five outpatient follow-up visits: 3 visits with a nurse or physician and 2 visits with a physiotherapist (Figure 1).

### The Intervention

**Figure 2:**
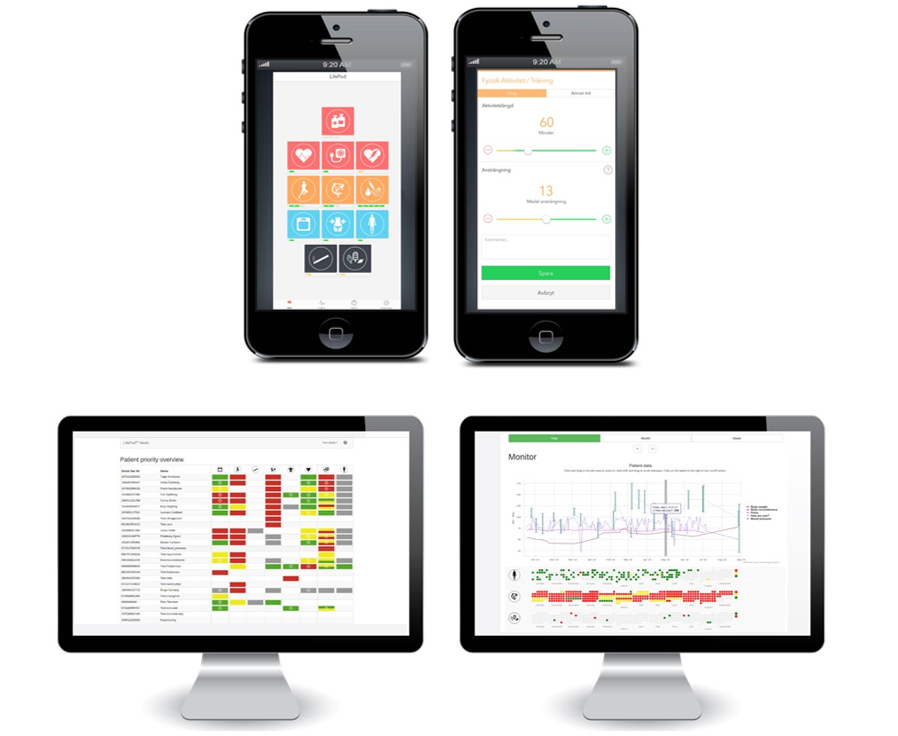
Screenshots of the LifePod patient interface (top) and medical interface (bottom). The software could be accessed through a smartphone, tablet, or computer.

Participants randomized to the intervention group of the study received access to the LifePod web-based mobile device app (Cross Technology Solutions AB) for the first 25 weeks of the cardiac rehabilitation program. The LifePod software contained two separate interfaces: one for the patient and one for the treating health care professionals (Figure 2). In the patient interface, the patient could log information about diet, physical activity and exercise, weight, heart rate, BP, and smoking, as well as symptoms and intake of medication. The patient could compare his or her own data to guideline-recommended targets and received automated positive feedback on healthy lifestyle choices as well as general recommendations on exercise, daily physical activity, and healthy diet. In the medical interface assessed by the treating cardiac rehabilitation staff, the system ranked patients, giving high priority to, for example, patients reporting chest pain or out-of-range BP measurements. The medical interface was reviewed twice a week by a nurse.

### Data Collection

Patient outcomes were derived from the SWEDEHEART registry. The SWEDEHEART registry is a nationwide quality registry that records baseline characteristics, treatments, follow-up, and outcome data of patients who have had an MI [9]. Data collection started at the time of index MI and continued at all follow-up visits, as shown in Figure 1. The time points for data collection were prespecified in accordance with standardized SWEDEHEART registry follow-up visits. Information was collected using standardized forms that can be downloaded from the SWEDEHEART registry webpage [24].

At the physiotherapist visits at 2 to 4 weeks and 4 to 6 months, a submaximal exercise test on a bicycle ergometer was performed [25,26]. During the test, heart rate, systolic BP (SBP), perceived exertion, dyspnea, and chest pain were registered according to the Borg rating of perceived exertion and category ratio-10 scales [27]. The test was discontinued at Borg rating of perceived exertion 17, dyspnea 7 on the Borg category ratio-10 scale, or other routine discontinuation criteria for exercise stress testing, including, for example, chest pain and fall in SBP. At the first visit, the patients received individualized physical activity and exercise recommendations and were invited to participate in exercise-based cardiac rehabilitation as a part of their comprehensive cardiac rehabilitation program.

The follow-up visits with a nurse or physician at approximately 2 weeks, 6 to 10 weeks, and 12 to 14 months focused on risk factors and lifestyle. Fasting plasma glucose (mmol/L), hemoglobin A_1c_ (mmol/mol), and plasma lipids (total cholesterol, LDL-cholesterol, high-density lipoprotein–cholesterol, and triglycerides; mmol/L) samples were drawn and analyzed using accredited methods at each hospital. BP was measured using a manual sphygmomanometer after a 5-minute rest with the patient in a sitting position. Weight (kg) was measured in light indoor clothing, and BMI (kg/m^2^) was calculated. Smoking status was self-reported. At baseline, 6 to 10 weeks, and 12 to 14 months, smoking abstinence was defined as being smoke-free for ≥1 month. At the 2-week visit, abstinence was defined as being abstinent at the time of the visit. Diet was evaluated using a 4-item questionnaire adapted from the national guidelines for the management of unhealthy lifestyle in the general population [28]. The questions aim to quantify the amount of vegetables, fruit, fish, and sweets consumed. Each question had 4 possible answers, giving 0 to 3 points. The scores for each question were subsequently added, forming the healthy diet index (0-12 points). Levels of physical activity were self-reported using 2 sets of questionnaires. Haskell questions on physical activity and exercise evaluated both the number of days during the last week [1-7] with at least 30 minutes of physical activity and the number of days during the last week [1-7] with at least 20 minutes of exercise training [29]. The Frändin-Grimby physical activity questionnaire aims to evaluate the level of physical activity a person achieved in the last week on a scale of 1 to 6, with 1 being hardly any physical activity and 6 being regular strenuous physical activity [30].

The data that support this study’s findings are available from the Uppsala Clinical Research Centre, Sweden. The primary responsibility for data monitoring, including data sharing, integrity, and security, was assigned to the principal investigator and local study coordinators. The trial was conducted according to Good Clinical Practice, and data were handled according to the Swedish Data Protection Authority and General Data Protection Regulations.

### Statistical Methods and Outcomes

With a power of 90%, a 2-sided significance level of .05, and a mean difference of at least 10 (SD 20) W among the groups, the estimated sample size needed was 50 participants in each group [22]. However, the expected loss of adherence to the web-based application led to the formation of an unequal allocation ratio of 1:2 in the usual care group versus intervention group.

The primary outcome was the change (δ value) in submaximal exercise capacity, measured in Watts, between 2 submaximal exercise tests performed at 2 to 4 weeks and at 4 to 6 months. The secondary outcomes were changes (δ values) in dietary habits and physical activity, as well as SBP and diastolic BP (DBP), total cholesterol, LDL-cholesterol, high-density lipoprotein–cholesterol, triglycerides, fasting plasma glucose, hemoglobin A_1c_, and BMI. The secondary outcomes also included smoking status and uptake and adherence to the web-based application. Uptake was defined as the proportion of patients who logged on to the patient interface at least once, and adherence was defined as the proportion of patients registering data at least twice per week on a weekly basis throughout the 25-week intervention period. An additional analysis on the difference in mortality and number of hospital readmissions during the trial period was performed. Baseline characteristics are presented as means (SDs) for normally distributed continuous variables, medians (IQR) for nonnormally distributed continuous variables, and as numbers and percentages for categorical variables. Variable distribution was assessed by visual inspection of histograms and Q–Q plots and by calculating skewness and kurtosis; *z* values between −1.96 and 1.96 were used to define the normally distributed interval. To compare outcome measures of continuous variables among the groups, a 2-tailed Student *t* test or a Mann–Whitney *U* test was performed. A univariate analysis of variance, adjusting for age, gender, weight, previous CHD, and smoking status at index MI was also performed. In the analysis of variance, the dependent variables were the measured outcomes, and the independent variables were the intervention and chosen covariates. For categorical variables, a chi-square test and a logistic regression analysis adjusting for the previously mentioned covariates were used. For within-group comparisons, a paired *t* test was used for normally distributed variables and a Wilcoxon signed-rank test for skewed data. For all analyses, an α level of .05, and 2-tailed testing was used. Individuals with missing data on covariates or outcome variables were excluded from the analysis (listwise exclusion). All data were analyzed by using the SPSS (version 25.0; IBM Corp).

## Results

### Participant Recruitment and Flow

**Figure 3:**
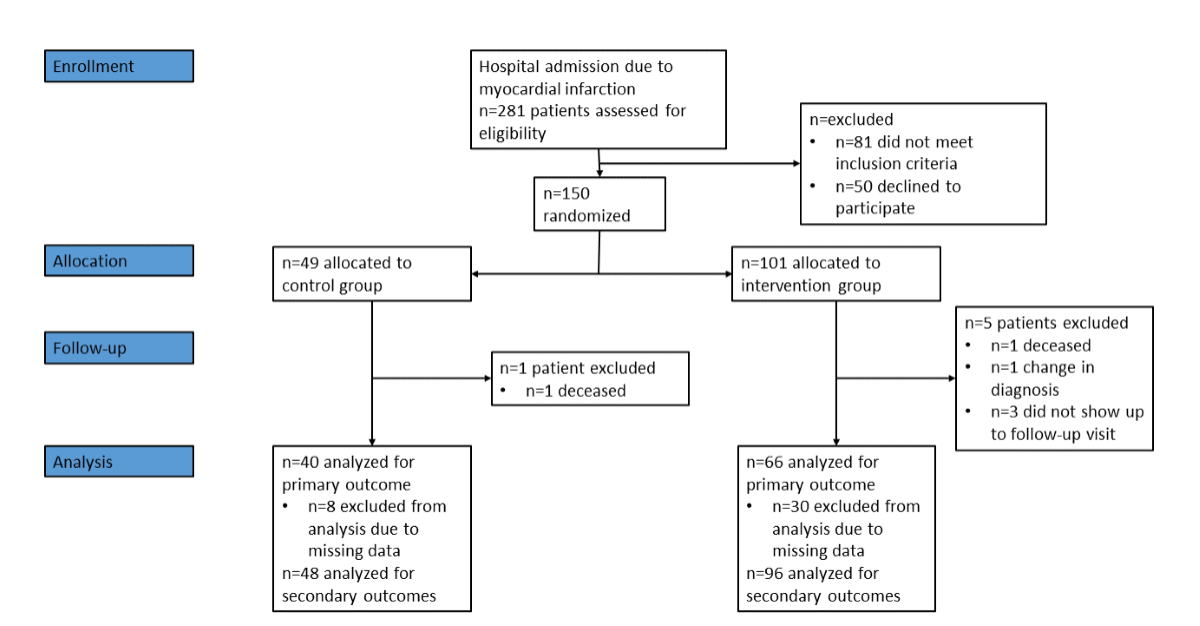
A flowchart displaying the recruitment process and flow of participants in the usual care and intervention groups.

Of the 281 patients assessed for eligibility, 150 (53.4%) consented and indicated intent to attend cardiac rehabilitation and, if allocated to the intervention group, use the web-based application (Figure 3). In total, 32.7% (49/150) of the patients were allocated to the control group and 67.3% (101/150) to the intervention group. At the end of the study period, 1 participant in each group of the study had died. For 1 patient, the index event diagnosis changed during follow-up from MI to cardiac amyloidosis, and the patient was excluded from the analysis. Of the 150 patients, 3 (2%) did not attend their follow-up visits at 12 to 14 months after MI and were classified as lost to follow-up.

Patient inclusion started in April 2016 and was finalized in April 2018. The follow-up period was completed in June 2019.

### Baseline Characteristics

Baseline characteristics are presented in Table 1. The intervention group had a slightly higher prevalence of cardiac comorbidities at baseline including a higher mean SBP and were more often taking cardioprotective medication compared with the usual care group. In addition, a proportionally higher number of patients in the intervention group had an ST elevation MI.

**Table 1:** Baseline characteristics.^a^

Parameters		Intervention (n=100)	Usual care (n=49)
Age (years), mean (SD)		60.0 (8.9)	61.1 (8.6)
Male gender, n (%)		84 (84)	36 (73)
Active smoker, n (%)		22 (22)	11 (22)
**Physiological measures**			
	SBP^b^ (mm Hg), mean (SD)	150.0 (27.6)	142.9 (25.5)
	DBP^c^ (mm Hg), mean (SD)	88.5 (14.6)	86.8 (14.8)
	Waist circumference (cm), mean (SD)	104.9 (12.9)	104.5 (13.9)
	Weight (kg), mean (SD)	86.3 (15.1)	85.3 (16.2)
	BMI (kg/m^2^), median (IQR)	27 (25-30)	27 (25-29)
**Laboratory measures**			
	Total cholesterol (mmol/L), mean (SD)	4.7 (1.1)	4.9 (1.1)
	LDL^d^-cholesterol (mmol/L), mean (SD)	2.8 (0.9)	3 (1.0)
	Triglycerides (mmol/L), median (IQR)	1.4 (0.9-2.1)	1.4 (1.0-1.9)
	HDL^e^-cholesterol (mmol/L), median (IQR)	1.2 (0.9-1.4)	1.2 (0.9-1.4)
	Fasting plasma glucose (mmol/L), median (IQR)	7.5 (6.4-9.2)	7.1 (6.2-8.9)
	HbA_1c_^f^ (mmol/mol), median (IQR)	38 (34.5-41.0)	39 (36.0-42.0)
**Previous disease, n (%)**			
	Ischemic heart disease (previous MI^g^, PCI^h^, or CABG^i^)	18 (18)	6 (12)
	Heart failure	5 (5)	0 (0)
	Diabetes mellitus	9 (9)	6 (12)
	Hypertension	42 (42)	17 (34)
**Medication on hospital admission, n (%)**			
	ACEi^j^ or ARB^k^	36 (36)	12 (24)
	Statins	24 (24)	5 (10)
	Acetylsalicylic acid	18 (18)	5 (10)
	β-blockers	19 (19)	7 (14)
**Medication at hospital discharge, n (%)**			
	ACEi or ARB	95 (95)	46 (93)
	Statins	100 (100)	47 (95)
	DAPT^l^	100 (100)	49 (100)
	β-blockers	89 (89)	43 (87)
**Type of MI, n (%)**			
	STEMI^m^	59 (59)	24 (48)
	NSTEMI^n^	41 (41)	24 (48)

^a^Data are presented as n (%), mean (SD), or median (IQR).

^b^SBP: systolic blood pressure.

^c^DBP: diastolic blood pressure.

^d^LDL: low-density lipoprotein.

^e^HDL: high-density lipoprotein.

^f^HbA_1c_: glycated hemoglobin A_1c_.

^g^MI: myocardial infarction.

^h^PCI: percutaneous coronary intervention.

^i^CABG: coronary artery bypass grafting.

^j^ACEi: angiotensin-converting enzyme inhibitor.

^k^ARB: angiotensin II receptor blocker.

^l^DAPT: dual antiplatelet therapy.

^m^STEMI: ST elevation myocardial infarction.

^n^NSTEMI: non-ST elevation myocardial infarction.

### Primary Outcome

Because of missing data at the first or second follow-up with a physiotherapist, only 82% (40/49) of participants in the usual care group and 66% (66/100) in the intervention group qualified for analyses of the primary outcome.

**Figure 4:**
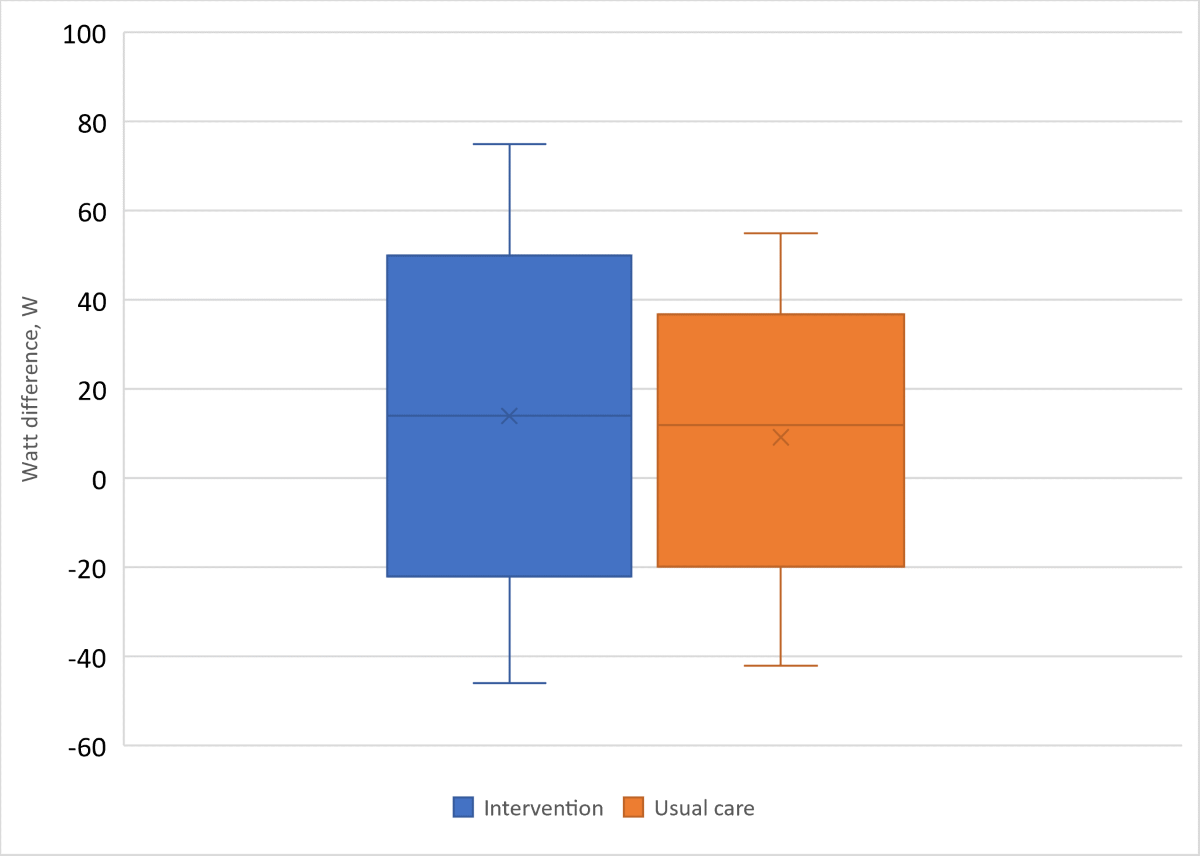
A boxplot of the change in submaximal exercise capacity in the intervention group and the usual care group. The difference in change in exercise capacity among the groups was nonsignificant.

Both groups increased their submaximal exercise capacity significantly between the first and second follow-up measurements (Figure 4). The intervention group increased their exercise capacity from 96.3 (SD 29.4) W to 110.8 W (SD 33.7 W; *P*<.001) and the corresponding values for the usual care group were 96.1 (SD 33.7) W to 106.5 W (SD 37.3 W; *P*<.001).

There was no significant difference in the change in exercise capacity between the intervention group and the usual care group (+14.4, SD 19.0 W vs +10.3, SD 16.1 W; crude 95% CI −11.2 to 3.0, *P*=.26; adjusted 95% CI −12.0 to 2.8, *P*=.22) at follow-up. The variation in the observed difference fell within the expected limits of +20 W to −20 W.

### Secondary Outcomes

**Figure 5:**
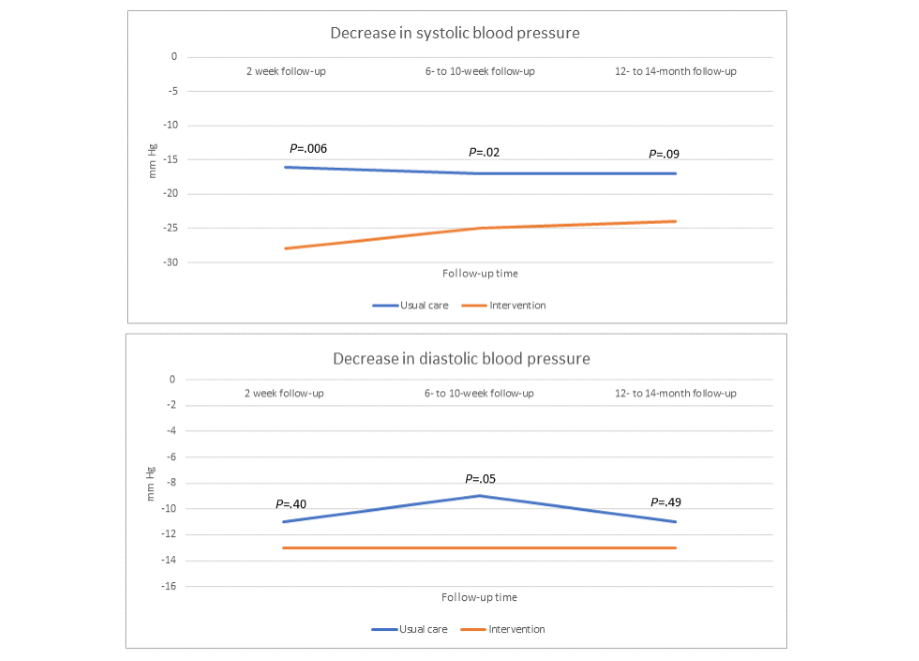
Decrease in systolic and diastolic blood pressure between baseline and follow-up visits (absolute values). *P* values are adjusted for gender, age, weight, previous coronary heart disease, and smoking status at the time of the index event.

The secondary outcomes are shown in Tables 2-4. Crude analyses showed that at the 2-week follow-up, there was a significantly larger decrease in SBP in the intervention group than that in the usual care group. SBP remained lower in the intervention group than in the usual care group for the remainder of the follow-ups; however, the difference among the groups was nonsignificant. In the adjusted analysis, the decrease in SBP was significantly larger in the intervention group between baseline and 2 weeks and for both SBP and DBP between baseline and 6 to 10 weeks (Figure 5).

**Table 2:** Secondary outcome measures at the 2-week follow-up.^a^

Characteristics		Intervention			Usual care			*P* value for difference crude		*P* value for difference adjusted^b^
		Values	Change from baseline	Values		Change from baseline				
**Risk factors**										
	SBP^c^ (mm Hg), mean (SD)	123.4 (17.4)	−27.7 (27.6)	127.3 (13.9)		−16.4 (24.1)	.02		.006	
	DBP^d^ (mm Hg), mean (SD)	76.0 (10.4)	−13.1 (13.0)	76.1 (10.2)		−11.1 (15.1)	.43		.40	
	BMI (kg/m^2^), median (IQR)	27.1 (24.6 to 31.7)	−0.3 (−0.9 to 0.5)	27.8 (24.8 to 30.8)		−0.1 (−0.7 to 0.6)	.34		.77^e^	
**Self-reported parameters, mean (SD)**										
	Vegetable consumption	2.2 (0.9)	0.4 (0.9)	2.2 (1.0)		0.4 (0.8)	.81		.94	
	Fruit consumption	2.3 (0.7)	0.7(1.0)	2.2 (0.9)		0.2 (1.0)	.03		.03	
	Fish consumption	1.9 (1.0)	0.8 (1.0)	1.7 (1.0)		0.4 (0.8)	.03		.02	
	Consumption of sweets	2.4 (0.8)	0.5 (0.9)	2.4 (0.7)		0.5 (0.9)	.99		.99	
	Healthy diet index	8.7 (2.1)	2.3 (2.1)	8.3 (2.1)		1.4 (2.3)	.05		.03	

^a^Numbers are presented as mean (SD), median (q1, q3), and *P* values. Crude and adjusted *P* values are shown for the differences in mean or median change (δ) between baseline and 2-week follow-up, comparing the intervention and usual care groups.

^b^Adjusted for gender, age, weight, previous heart disease, and smoking status at the time of the index event.

^c^SBP: systolic blood pressure.

^d^DBP: diastolic blood pressure.

^e^Not adjusted for weight.

The healthy diet index scores improved to a significantly larger extent between baseline and the 2-week follow-up in the intervention group than in the usual care group, mostly because of an increase in the consumption of fish and fruit (Table 2). However, this improvement was not sustained for the rest of the follow-up period (Tables 3 and 4).

There were no significant differences in self-reported physical activity among the groups. Results from the Haskell questions on physical activity and exercise [29] showed a mean change in number of days of performing at least 30 minutes of physical activity between baseline and the first follow-up with a physiotherapist at 2 to 4 weeks, which was 3.1 days (SD 2.2 days) for the intervention group and 2.5 days (SD 2.3 days) for the usual care group (adjusted *P*=.13). The corresponding numbers between baseline and the second follow-up with a physiotherapist at 4 to 6 months were 2.6 days (SD 2.6 days) and 1.8 days (SD 2.2 days) for the intervention and control groups, respectively (adjusted *P*=.08). The mean change in the number of days performing at least 20 minutes of exercise training between baseline and the first follow-up with a physiotherapist was 0.2 days (SD 2.0 days) for the intervention group and 0.1 days (SD 1.7 days) for the control group (adjusted *P*=.76), and the corresponding numbers between baseline and the second follow-up were 1.2 days (SD 2.2 days) and 1.3 days (SD 2.4 days) for the intervention and control groups, respectively (adjusted *P*=.79).

According to the Frändin-Grimby physical activity questionnaire [30], the mean change in the level of physical activity a person achieved in the last week between baseline and the first follow-up with a physiotherapist at 2 to 4 weeks was 0.1 (SD 1.0) point in the intervention group and 0.3 (SD 1.0) point for the usual care group (adjusted *P*=.21). The mean change between baseline and the second follow-up visit with a physiotherapist at 4- to 6-month visit was 0.6 (SD 1.2) in the intervention group and 0.8 (SD 1.1) in the usual care group (adjusted *P*=.46).

A total of 33 smokers were included in the trial, 22 in the intervention group and 11 in the usual care group. At the 2-week follow-up, 64% (14/22) of smokers in the intervention group and 55% (6/11) of smokers in the usual care group reported being abstinent from smoking (adjusted *P*=.76). At 6 to 10 weeks, the numbers were 64% (14/22) vs 46% (5/11; adjusted *P*=.24), and at 12 to 14 months, they were 55% (12/22) vs 36% (4/11; adjusted *P*=.74), for the intervention and control groups, respectively.

**Table 3:** Secondary outcome measures at the 6- to 10-week follow-up.^a^

Characteristics			Intervention					Usual care					*P* value for difference crude			*P* value for difference adjusted^b^	
			Values		Change from baseline		Values			Change from baseline							
**Risk factors**																	
	SBP^c^ (mm Hg), mean (SD)	123.6 (14.8)		−25.3 (27.4)		127.1 (13.3)			−16.5 (27.4)		.08			.02			
	DBP^d^ (mm Hg), mean (SD)	75.3 (10.0)		−13.4 (15.6)		77.8 (8.7)			−9.1 (13.4)		.11			.05			
	BMI (kg/m^2^), median (IQR)	26.3 (24.0 to 29.0)		−0.3 (−1.1 to 0.3)		27.0 (24.3 to 29.9)			−0.2 (−1.3 to 0.3)		.77			.39^e^			
	Total cholesterol (mmol/L), mean (SD)	3.3 (0.8)		−1.5 (1.1)		3.3 (0.6)			−1.6 (0.9)		.48			.37			
	LDL^f^-cholesterol (mmol/L), mean (SD)	1.5 (0.6)		−1.4 (0.9)		1.5 (0.4)			−1.5 (0.8)		.28			.24			
	HDL^g^-cholesterol (mmol/L), median (IQR)	1.1 (0.9 to 1.5)		0.1 (−0.1 to 0.2)		1.2 (1.0 to 1.5)			0.1 (−0.1 to 0.3)		.80			.93			
	Triglycerides (mmol/L), median (IQR)	1.0 (0.8 to 1.5)		−0.3 (−0.8 to 0.0)		1.1 (0.8 to 1.4)			−0.3 (−0.7 to 0.0)		.93			.99			
	Fasting plasma glucose (mmol/L), median (IQR)	6.1 (5.8 to 6.8)		−1.4 (−3.3 to −0.3)		6.2 (5.6 to 7.0)			−0.9 (−2.8 to −0.2)		.81			.47			
**Self-reported parameters, mean (SD)**																	
	Vegetable consumption	2.2 (0.7)		0.3 (0.8)		2.2 (0.7)			0.4 (1.1)		.74			.65			
	Fruit consumption	2.3 (0.7)		0.6 (1.0)		2.3 (0.7)			0.5 (1.1)		.29			.36			
	Fish consumption	2.1 (1.0)		0.9 (1.1)		2.0 (0.9)			0.8 (1.1)		.40			.18			
	Consumption of sweets	2.3 (0.9)		0.3 (0.9)		2.3 (0.9)			0.5 (1.1)		.27			.22			
	Healthy diet index	8.9 (2.0)		2.2 (2.3)		8.8 (1.7)			2.1 (2.6)		.82			.77			

^a^Numbers are presented as mean differences (SD), median differences (IQR), and *P* values. Crude and adjusted *P* values are shown for the differences in mean or median change (δ) between baseline and 6- to 10-week follow-up, comparing the intervention and usual care groups.

^b^Adjusted for gender, age, weight, previous heart disease, and smoking status at the time of the index event.

^c^SBP: systolic blood pressure.

^d^DBP: diastolic blood pressure.

^e^Not adjusted for weight.

^f^LDL: low-density lipoprotein.

^g^HDL: high-density lipoprotein.

**Table 4:** Secondary outcome measures at 12- to 14-month follow-up.^a^

Characteristics			Intervention				Usual care				*P* value for difference crude		*P* value for difference adjusted^b^
			Values		Change from baseline		Values		Change from baseline				
**Risk factors**													
	SBP^c^ (mm Hg), mean (SD)	126.9 (16.0)		−24.0 (31.1)		126.5 (13.7)		−17.0 (28.3)		.22		.09	
	DBP^d^ (mm Hg), mean (SD)	76.1 (10.9)		−12.6 (17.6)		75.7 (9.2)		−11.3 (17.1)		.69		.49	
	BMI (kg/m^2^), median (IQR)	26.4 (23.6 to 30.1)		−0.3 (−1.3 to 1.0)		27.1 (24.8 to 29.1)		−0.5 (−1.2 to 0.8)		.57		.35^e^	
	Total cholesterol (mmol/L), mean (SD)	3.5 (0.9)		−1.3 (1.2)		3.5 (0.8)		−1.5- (1.1)		.41		.30	
	LDL^f^-cholesterol (mmol/L), mean (SD)	2.1 (1.0)		−1.2 (1.1)		2.0 (0.9)		−1.4 (1.0)		.20		.21	
	HDL^g^-cholesterol (mmol/L), median (IQR)	1.2 (1.0 to 1.5)		0.1 (0.0 to 0.3)		1.3 (1.0 to 1.5)		0.1 (0.0 to 0.3)		.89		.77	
	Triglycerides (mmol/L), median (IQR)	1.0 (0.7 to 1.4)		−0.3 (−0.9 to 0.0)		1.2 (0.8 to 1.5)		−0.3 (−0.8 to 0.8)		.48		.98	
	Fasting plasma glucose (mmol/L), median (IQR)	6.0 (5.6 to 6.7)		−1.5 (−3.3 to −0.2)		6.0 (5.5 to 6.5)		−1.4 (−2.7 to 2.0)		.45		.48	
	HbA_1c_^h^ (mmol/mol), median (IQR)	40.0 (36.0 to 43.0)		1.0 (−0.2 to 3.0)		38.0 (37.0 to 43.0)		2.0 (−2.0 to 3.8)		.68		.40	
**Self-reported parameters, mean (SD)**													
	Vegetable consumption	2.1 (0.8)		0.3 (1.0)		2.0 (0.8)		0.2 (0.7)		.67		.77	
	Fruit consumption	2.0 (0.8)		0.3 (0.8)		2.2 (0.8)		0.3 (0.9)		.92		.99	
	Fish consumption	1.8 (1.1)		0.6 (1.0)		2.0 (0.9)		0.7 (1.0)		.70		.66	
	Consumption of sweets	2.3 (0.8)		0.4 (1.0)		2.1 (0.9)		0.3 (1.1)		.45		.37	
	Healthy diet index	8.2 (2.1)		1.6 (2.2)		8.2 (1.8)		1.4 (2.2)		.69		.45	

^a^Numbers are presented as mean differences (SD), median differences (q1, q3), and *P* values. Crude and adjusted *P* values are shown for the differences in mean or median change (δ) between baseline and 12- to 14-month follow-up, comparing the intervention and usual care groups.

^b^Adjusted for gender, age, weight, previous heart disease, and smoking status at the time of the index event.

^c^SBP: systolic blood pressure.

^d^DBP: diastolic blood pressure.

^e^Not adjusted for weight.

^f^LDL: low-density lipoprotein.

^g^HDL: high-density lipoprotein.

^h^HbA_1c_: glycated hemoglobin A_1c_.

### Uptake and Adherence

Attendance to the cardiac rehabilitation program was generally high. A total of 93% (93/100) of patients in the intervention group attended the first follow-up visit with a physiotherapist, and 71% (71/100) attended the second. The corresponding percentages for the usual care group were 98% (48/49) and 86% (42/49). However, only 66% (66/100) of patients in the intervention group and 82% (40/49) of patients in the usual care group performed a submaximal exercise test at both visits.

A total of 92% (92/100) of patients in the intervention group attended the 6- to 10-week follow-up and 96% (96/100) attended the 12- to 14-month follow-up, and the corresponding numbers for the usual care group were 98% (48/49) for both follow-up visits.

**Figure 6:**
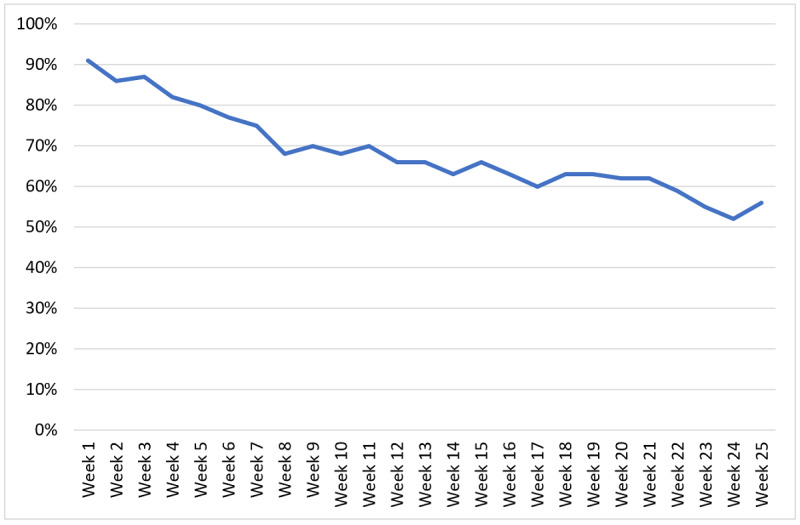
Percentage of patients logging data to the web-based application at least twice weekly.

Uptake to the web application was 86.1% (87/101). Adherence (the proportion of the 87 patients who continued to log data at least twice per week) declined during the trial period, from its highest of 91% (79/87) at week 1 to 56% (49/87) at week 25 (Figure 6).

The most frequent parameters to be logged to the web-based application by patients in the intervention group were intake of medication and consumption of vegetables, followed by physical activity (Multimedia Appendix 1).

### Additional Analysis

There was no difference in the frequency of deaths due to any cause (n=1 in each group, *P*=.62). The frequencies of rehospitalizations during the 12- to 14-month follow-up period were also similar among the groups. In total, 22% (22/100) of the patients in the intervention group and 27% (13/49) of the patients in the usual care group were rehospitalized due to any cause (*P*=.58), and 8% (8/100) versus 4% (2/49) of the patients were hospitalized due to ischemic heart disease (*P*=.35).

## Discussion

### Principal Findings

In our study, evaluating the effects of a web-based application as an addition to usual care cardiac rehabilitation as compared with usual care alone, we found that there was a trend toward better exercise capacity in the intervention group; however, this was not statistically significant. On the other hand, the patients receiving access to the web-based application had a significantly reduced SBP at the first 2 follow-up visits, a reduced DBP at the second follow-up visit, and an initial increase in intake of fish and fruit. Although cardiac rehabilitation attendance was high in both groups, adherence to the web-based application declined over time.

After the baseline exercise test, all patients in our study received individualized physical activity recommendations and an individualized exercise prescription and were invited to participate in exercise-based cardiac rehabilitation based at the cardiac rehabilitation center. Those in the intervention group were, in addition, advised to rate their perceived exertion and time spent in exercise and log the information on the web-based application, after which they would receive automated positive feedback. Although attendance to physiotherapy follow-up visits was high, not all patients completed the submaximal exercise tests. The reasons for noncompletion were not officially documented, but according to clinical experience, reasons often include common cold, leg or joint pain, and perceived inability to exercise. During follow-up, both groups had a significant improvement in submaximal exercise capacity and, although not significant, there was a 4.1 W difference among the groups in favor of the intervention group. Increased physical activity levels using telehealth in cardiac rehabilitation have been demonstrated in previous studies. A recent meta-analysis showed that this applied especially to studies where telehealth was used as an adjunct to comprehensive cardiac rehabilitation [16]. The web-based application in our study did not specifically provide feedback on adherence to the exercise-based cardiac rehabilitation program but rather to increase physical activity levels in general, which may perhaps partly explain the lack of significant differences in exercise capacity among the groups.

The benefit of the combination of involvement of health care professionals with eHealth has been shown to be beneficial in studies addressing cardiovascular risk factors [31-33]. For example, a recent RCT found that participants using an exercise-focused smartphone app had better fitness levels (measured by maximal oxygen consumption or VO_2peak_) compared with usual care after a follow-up at the 1-year postcardiac rehabilitation [32]. The information that participants logged into the smartphone app was monitored by a physiotherapist who provided personal feedback. Adherence to the smartphone app was high, and participants felt that they reported to an individual involved in their care rather than a database or a robot. This knowledge can be used in the design of future studies aiming to increase the exercise-based cardiac rehabilitation adherence with the overall aim of increasing patients’ physical fitness.

Most patients who had an MI were already on a regime of cardioprotective medication when discharged from the hospitals (Table 1). Despite this, a significant difference in BP control was observed among the groups, in favor of the intervention group. In our crude analysis, patients in the intervention group had a significantly larger SBP decrease between baseline and the 2-week follow-up. A numerical but nonsignificant difference among the groups then remained throughout the trial period. After adjusting for relevant covariates, the observed differences in BP control increased, in favor of the intervention group, and included a significant difference in SBP and DBP decrease between baseline and the 6- to 10-week follow-up. Patients in the intervention group achieved an 11.3 mm Hg larger decrease in SBP between baseline and the 2-week follow-up compared with patients in the usual care group, the difference being 8.8 mm Hg at the 6 to 10-week follow-up and 7.0 mm Hg at the 12- to 14-month follow-up. The choice of covariates was based on observed differences in baseline characteristics, which indicated a higher comorbidity in the intervention group. As such, not adjusting for baseline differences could have masked a difference among the groups. Hypertension is a predominant risk factor for CHD morbidity and mortality [7]. Previous meta-analyses have shown that a 10 mm Hg reduction in SBP reduces the risk of future CHD events by 22%, irrespective of the method of BP reduction [34]. Given the magnitude of the observed decrease in BP in our study, the difference is likely to have some clinical benefit. An improvement in reaching BP goals when having access to a web-based application as an aid in cardiac rehabilitation is in line with previous studies [11-13,20,35]. For example, Widmer et al [35] also demonstrated a substantial BP difference, with patients who after a percutaneous coronary intervention received digital health intervention (web-based or smartphone-based) as a complement to traditional cardiac rehabilitation having significant improvements in SBP (−10.8 mm Hg, *P*<.001 vs −6.1 mm Hg, *P*=.36) at a 3-month follow-up. Because intake of medication was the most frequently logged parameter in the web-based application, one possible reason for improved BP values in the intervention group might be an increased compliance to antihypertensive medication. Increased adherence to medication has been demonstrated in a previous study on a web-based application in cardiac rehabilitation [36]. In addition, increased patient engagement with self-monitoring has been shown to improve BP control [37,38]. The difference in BP among the groups declined throughout the trial period. One reason might be that adherence to behavioral recommendations has been seen to decline over time in cardiac rehabilitation, or it could have something to do with decreasing adherence to the web-based application which is a known methodological challenge within eHealth [11,39]. As there was no measurement done at the end of the 25-week intervention period, we do not know exactly when the differences attenuated.

We also observed a significant beneficial effect on healthy dietary choices. During the first 2 weeks of follow-up, patients in the intervention group had a significantly higher score on the health diet index and higher intake of fish and fruit. Following a healthy diet rich in healthy oils and plant-based food has been shown to reduce cardiovascular events [40]. Previous studies on eHealth in cardiac rehabilitation have shown both improvement and no effect on dietary habits [15,16,41]. Here, the data were self-reported, which has considerable limitations, and the results should be interpreted with caution [42].

Smoking is a leading cause of preventable death globally and is a strong risk factor for CHD [7]. There was a numerical difference in the number of smokers who reported being abstinent at the follow-up visits, but owing to the low number of smokers in our study population, the differences were not statistically significant. In addition, whether the numerical difference was due to the intervention or chance finding cannot be stated. However, telehealth interventions in cardiac rehabilitation have been shown to reduce the likelihood of smoking by 23%, giving promise to using eHealth as a tool to tackle this important risk factor [16].

### Adherence to eHealth

eHealth has the potential to improve participation and adherence to cardiac rehabilitation programs by including patients more actively in their own care and increasing flexibility and accessibility [21]. In our study, although uptake to the web-based application was high, adherence declined over time, with just over half of the users logging data at least twice weekly at the end of the 25-week intervention period. The main reason for limiting the intervention time to 25 weeks was to harmonize the intervention length with usual care, as most usual care cardiac rehabilitation interventions take place during the first 6 months after MI (nurse and physiotherapist visits, exercise training sessions, and patient education). In addition, patients usually have the highest level of motivation for lifestyle changes during the first months after MI [39]. The last reason was that adherence to eHealth interventions in cardiac rehabilitation has been shown to attenuate over time (weeks to months) [11,43]. In our study, reported reasons for stopping the use of the web-based application were mostly related to stress, some experienced a lack of feedback and some experienced too much feedback. User attrition in studies on eHealth is a well-known methodological challenge, with attrition rates reaching up to 60% to 80% [43]. High attrition rates can make it difficult to measure an intervention’s effect and subsequently threaten both internal and external study validity [44,45]. Van der Mispel et al [44] studied user and website characteristics related to attrition. They demonstrated that attrition was higher for men and younger adults, as well as for less interactive components of the studied application. Buys et al [46] reported a general interest in technology-enabled home-based cardiac rehabilitation among patients with cardiovascular disease. Their study demonstrated that patients with different characteristics were interested in different types of technology-based cardiac rehabilitation; for example, older patients were more interested in web-based options, and younger patients were more interested in app-based cardiac rehabilitation. They also looked at which parts of cardiac rehabilitation patients wanted to have technology-based options, including ideas on exercise, healthy meals, and stress management. This indicates that even if technology-based cardiac rehabilitation aims to make traditional programs more flexible and individualized, even the technology platforms need to be adjusted to individual needs for optimum effect.

### Strengths and Limitations

In our study, we used data collected through standardized protocols from the SWEDEHEART registry [9,47]. The registry is well established in Swedish cardiac rehabilitation centers and is used daily by personnel. At the time of the study, SWEDEHEART nationwide coverage was >75%, registering eligible cases from 97% of Swedish hospitals. Data quality is regularly monitored, showing >95% agreement with data from hospital records [48]. Using SWEDEHEART prespecified time points for follow-up and procedures provided a standardization, which otherwise can be a challenge in multicenter trials. Cardiac rehabilitation program attendance was high, and few participants were lost on follow-up.

The study was unblinded. Although blinding is preferred in RCTs, it is difficult to blind patients and health care providers in eHealth interventions. The follow-up data do not include information on the use of commercial web-based applications by the patients in the usual care group. The age limit considered in this study may restrict generalizability to all age groups. There might have been some selection bias, where more motivated patients would be more likely to agree to participate in a study using digital technology, requiring active participation. When interpreting the results, one should keep in mind the potential sources of inaccuracy in point BP measurements, which is a known source of bias in the clinical setting [49].

### Conclusions

Digital technologies provide new opportunities in health care. Our results add to existing evidence and suggest that complementing comprehensive cardiac rehabilitation programs with a web-based application may positively affect risk factor outcomes and lifestyle, including BP and dietary choices. In addition, web-based technologies can be used to make cardiac rehabilitation programs more flexible and individualized, but further efforts should be invested to find ways to improve patient adherence to the platforms.

## Appendix

Multimedia Appendix 1 Percentage of reports per parameter in the web-based application.

Multimedia Appendix 2 CONSORT-eHEALTH checklist (V 1.6.1).

## Abbreviations

BP: blood pressure
CHD: coronary heart disease
DBP: diastolic blood pressure
LDL: low-density lipoprotein
MI: myocardial infarction
RCT: randomized controlled trial
SBP: systolic blood pressure
SPIRIT: Standard Protocol Items: Recommendations for Interventional Trials
SWEDEHEART: Swedish Web-System for Enhancement and Development of Evidence-Based Care in Heart Disease Evaluated According to Recommended Therapies
