# Supplementary material for: Effect of a Lifestyle-Focused Web-Based Application on Risk Factor Management in Patients Who Have Had a Myocardial Infarction: Randomized Controlled Trial
Source: J Med Internet Res. 2022 Mar 31;24(3):e25224. doi: 10.2196/25224 (PMC9015765; doi:10.2196/25224)
Supplement: Multimedia Appendix 1 [file jmir_v24i3e25224_app1.docx]

Multimedia appendix 1.

*Figure 7. Percentage of reports per parameter in the web-based application.*
